# Buildings Contemplated

**Published:** 1914-07-18

**Authors:** 


### Buildings Contemplated.

Battersea, London, S.W.—Mr. Edwin T. Hall is the architect of the new out-patient department of the Anti-Vivisection Hospital, the foundation-stone of which has been formally laid.

Beccles.—The governors of Beccles Hospital are considering the question of building a new hospital, estimated to cost £5,000.

Birkenhead.—Plans for extensions and alterations to the Borough Hospital have been prepared. The estimated expenditure involved is £12,000.

Bootle.—The governors of the Borough Hospital have under consideration an improvements scheme, which includes the erection of a nurses' home and an extension of the buildings. The architects are Messrs. Hobbs and Black, of Liverpool.

Guisborough.—The Guardians have decided to engage an architect to prepare plans for the reconstruction of the workhouse.

Luton.—The Sanitary Committee has recommended the Town Council to apply to the Local Government Board for sanction to borrow £6,410 to defray the cost of extending the Spittlesea Hospital.

Morpeth.—It is proposed to build a joint isolation hospital for the districts of Morpeth, Ashington, and Newbiggin, at a cost of £30,000.

Paddington.—The Guardians have approved plans for a new nurses' home. The estimated cost is £10,330.

Pontypool.—Tenders are invited for the erection of a nurses' home at Pontypool for the Pontypool and District Hospital. Quantities may be obtained of Messrs. Longher and Co., Bank Chambers, Pontypool, Mon.

Ruthin.—The Guardians have resolved to erect a new infirmary at an estimated cost of £4,500, and tenders for the work are invited.

Tooting Bec, S.W.—The Metropolitan Asylums Board are inviting tenders for the extension of Tooting Bec Asylum. Full particulars may be obtained at the offices of the Board, Embankment, E.C., and drawings and specifications may be seen at the office of the architect, Mr. T. W. Aldwinckle, F.R.I.B.A., 20 Denman Street, London Bridge, S.E.

Trecynon.—The Aberdare Urban District Council have decided upon a site at Trecynon for their new isolation hospital. The estimated cost is £10,000.

